# Assessment of Synthetic Membranes for Artificial Blood Feeding of Culicidae

**DOI:** 10.3390/insects12010015

**Published:** 2020-12-29

**Authors:** Luciana S. Dias, Jonatas C. Caldeira, Luiz G. S. R. Bauzer, José B. P. Lima

**Affiliations:** 1 Laboratório de Fisiologia e Controle de Artrópodes Vetores, Instituto Oswaldo Cruz, Fiocruz, Rio de Janeiro 21040-360, Brazil; lucianad@ioc.fiocruz.br (L.S.D.); jonatas.caldeira@gmail.com (J.C.C.); jbento@ioc.fiocruz.br (J.B.P.L.); 2 Laboratório de Entomologia, Instituto de Biologia do Exército, Rio de Janeiro 209911-270, Brazil

**Keywords:** *Aedes aegypti*, *Culex quinquefasciatus*, artificial blood feeding, synthetic membranes, animal welfare

## Abstract

**Simple Summary:**

The maintenance of mosquito colonies in the laboratory requires a blood supply so that females can mature their oocytes and perform oviposition. Due to current bioethical parameters, the direct use of animals is being replaced by artificial blood feeding using equipment that requires the use of synthetic membranes to simulate animal skin. These membranes separate blood stored in a heated container from the external environment. The membranes need to be thin enough to allow for mosquitoes to feed, and strong enough that they do not easily break. In this study, the efficiency of collagen and latex in the artificial feeding of mosquitoes of the *Aedes aegypti* and *Culex quinquefasciatus* species was evaluated and compared with Parafilm^®^, a standard membrane that is frequently used for this purpose. Important aspects of the feeding and reproduction of these insects were considered. Collagen was almost as efficient as Parafilm^®^ was. Latex, on the other hand, negatively affected several of the analyzed aspects. We concluded that, although collagen is more resistant and easier to handle, Parafilm^®^ is the most efficient among the three evaluated membranes for mosquito artificial blood feeding.

**Abstract:**

Potential pathogen transmission through hematophagy in Culicidae is a major public-health problem, and several studies have been performed to better understand this phenomenon. Research on these insects often requires the maintenance of colonies in the laboratory. Due to the hematophagic habits of these organisms, blood must be provided in order to guarantee the reproduction of individuals that constitute the colonies. Some species of mammals and birds are used as a direct blood source in many laboratories. Due to current bioethical parameters, the direct use of animals has been replaced by artificial blood feeding by using synthetic membranes to simulate animal skin. In this study, the efficiency of collagen and latex in the artificial feeding of mosquitoes of the *Aedes aegypti* and *Culex quinquefasciatus* species was evaluated and compared with Parafilm^®^, a standard membrane that is frequently used for this purpose. Important aspects of the feeding and reproduction of these insects were considered. For both species, latex showed the poorest performance. Collagen membrane performed well in most parameters, but was not as efficient as Parafilm^®^ for fecundity in *Aedes aegypti*, and for the percentage of engorged females in *Culex quinquefasciatus*. We concluded that, although collagen is more resistant and easier to handle, Parafilm^®^ was the most efficient among the three evaluated membranes for the artificial blood feeding of mosquitoes.

## 1. Introduction

Hematophagy is a feeding habit of several families of the Diptera order. In Culicidae, it is carried out only by females, being a crucial factor in their biology, especially regarding to the reproductive aspect, since oocyte maturation and oviposition only occur after a blood meal [1]. The ability of Culicidae vectors to transmit pathogens during hematophagy is a major public-health problem [2]; several studies [3,4,5] were conducted to better understand this phenomenon. Many of these studies require the establishment of laboratory mosquito colonies that require a blood supply for maintenance [6,7]. Obtaining blood from animals in vivo is still a standard procedure, but this must comply with the current laws, protocols, and regulations approved by ethics committees and regulatory bodies [8]. Currently, bioethical parameters are being followed that include the three R principles: reduction, refinement, and replacement, related to animal welfare [9,10]. This led the scientific community to replace traditional methods, which include the use of animals applied to teaching and scientific research, with alternative methods. The search for new technologies will eventually restrict the use of animals [6,7,11].

The artificial blood feeding of mosquitoes is an alternative method for the use of animals in vivo. The artificial feeding of Culicidae is increasingly employed for the maintenance of these insects in laboratories. Most artificial-feeding studies described in the literature have an approach related to the experimental infection of colonies of, in most cases, small-scale feeding [8,12,13,14]. However, in recent years, some laboratories have started the large-scale rearing of mosquitoes for control approaches such as the sterile-insect technique. Those laboratories use Hemotek^®^, a specific equipment for the artificial blood feeding of insects (Hemotek Ltd., Blackburn, UK) [8,15,16]. There are cheaper alternatives to Hemotek^®^, such as the use of a water bath to heat glass feeders containing blood. All equipment used in artificial blood-feeding processes requires the use of synthetic membranes that separate blood stored in a heated container from the external environment. These membranes must be punctured by mosquitoes so that the blood can be ingested. Therefore, it is essential to choose a membrane that does not interfere with factors associated with the success of insect maintenance in the laboratory, such as the amount of ingested blood, fecundity, and egg hatching rate. Different membranes have been used in artificial-feeding procedures in an attempt to simulate the skin of vertebrate animals. Some studies indicated mosquitoes’ predilection for natural membranes, but there are records that other synthetic membranes, such as collagen gut and Parafilm^®^, were successfully used [17,18,19,20,21,22].

Parafilm^®^, when used in artificial feeding, is a membrane that is difficult to handle and, in most cases, cannot be reused. In addition, this membrane can very easily rupture when stretched, compromising the entire artificial-feeding process, making artificial feeding difficult in a laboratory routine. Thus, the evaluation of other types of membranes is necessary for the improvement of this technique.

The efficiency of collagen and latex in the artificial feeding of mosquitoes of the *Aedes aegypti* and *Culex quinquefasciatus* species was evaluated in this study. Important aspects of the survival and reproduction of these insects were considered, such as the percentage of feeding, the volume of ingested blood, fecundity, and egg-hatching rate. Collagen and latex were compared with Parafilm^®^, a frequently used membrane in artificial blood feeding, despite it being prone to rupture. It is essential to find an ideal membrane that does not interfere with the feeding and reproductive aspects of hematophagous insects, as it can help to reduce costs and in the standardization of the type of membrane for the maintenance and conservation of Culicidae colonies on a large scale. It is also important for the maintenance of global colony populations at desirable and stable levels according to the demands of each laboratory.

## 2. Materials and Methods

### 2.1. Larva Rearing and Adult Maintenance

Larvae of *Aedes aegypti* were reared in 27 × 19 × 7 cm plastic bowls containing 1 L of dechlorinated water and 1 g of cat food (Friskies^®^, Purina^®^, São Paulo, Brazil). Bowls were covered with a mesh lid. Cat food was replenished every three days. Larvae were reared in incubators adjusted to a temperature of 26 ± 1 °C, relative humidity of 70% ± 10%, and a photoperiod of 12 h light and 12 h dark (L:D 12:12 h). The same procedure was adopted for *Culex quinquefasciatus* larvae. Pupae were transferred to 50 mL plastic cups with the aid of a Pasteur plastic pipette and stored in cardboard cages measuring 16.5 cm in diameter × 17.5 cm in height. The resulting adults were fed ad libitum with 10% sucrose solution and kept under the same environmental conditions as those in which the larvae were kept.

### 2.2. Membranes

Collagen and a latex glove were evaluated and compared with Parafilm^®^ (Pechinery Plastic Packaging, Neenah, WI, USA). Parafilm^®^ was cut to 5 × 5 cm size and stretched to 15 × 15 cm, thereby reducing the thickness of the membrane in order to facilitate feeding according to a procedure standardized by Dias et al. [7]. The latex-glove membrane was cut to 3 × 3 cm size and washed in dechlorinated tap water to remove the powder covering the inner part of the glove. Collagen was cut, stretched up to 9 × 9 cm, and applied to the feeder base. Rubber elastics were used to attach the membranes to the feeders.

### 2.3. Feeding

**Figure 1:**
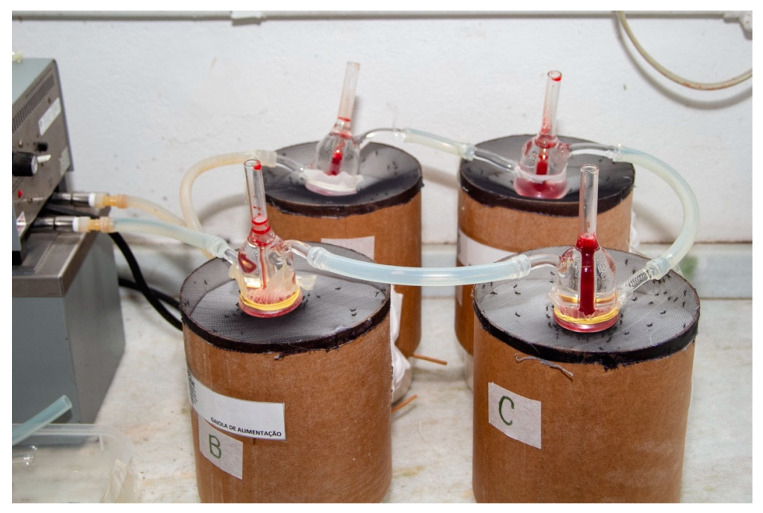
Artificial blood-feeding system for mosquitoes: four cages with retractable bottoms. Feeders filled with human blood are on top of the cages.

Human blood was obtained from the disposal of the blood bank of the Instituto de Biologia do Exército (IBEx), Rio de Janeiro, Brazil. On 9 December 2015, the Research Ethics Committee of Fiocruz authorized the experiments carried out using human blood (protocol number 1.358.833). This was in accordance with the Declaration of Helsinki of 1975 (https://www.wma.net/what-we-do/medical-ethics/declaration-of-helsinki/), revised in 2013. Figure 1 shows the feeding system.

Both species of mosquitoes were placed in cylindrical cardboard cages measuring 16.5 cm in diameter by 17.5 cm in height. The cages’ upper part was covered with a mesh, and they had a retractable bottom. Each cage contained 100 females. During feeding, each cage bottom was moved upwards towards the mesh in a way that reduced flight space and facilitated the perception of heat of the feeders by the females. The used feeders consisted of conical segments made of jacketed glass connected by an external circuit filled with water that circulated through the glasses. The arrangement prevented the contents (blood) of the internal reservoir from coming into contact with the external reservoir where the water circulated [18,23]. The system kept the blood at the desired temperature of 38 °C by circulating hot water. The opening of the bottom base was covered with one of the three types of evaluated membranes. Five to seven days after the emergence of adults, the artificial-feeding system was coupled to cages containing the adults. Twenty-four hours before delivering the blood meal, 10% sucrose solution was removed from the cages. For each membrane, six replicates of the experiment were assembled per species (two cages per day on three different days). In all replicates, the meal lasted from 30 to 40 min. Four cages were fed at a time, and the different membranes were randomly arranged to avoid experiment bias.

### 2.4. Percentage of Engorged Females

This experiment was to compare success in feeding according to the type of used membrane. After the feeding procedure, the fully engorged females of both species were counted by visual inspection, and their percentage was computed.

### 2.5. Quantification of Ingested Blood

This experiment aimed to determine the amount of ingested blood by females of *Aedes aegypti* and *Culex quinquefasciatus* according to the type of used membrane. Shortly after the feeding process described in Section 2.3, engorged female mosquitoes were anesthetized for 30 s in tubes containing ethyl acetate. Two groups were formed for weighing. The first group consisted of three pools, each containing 10 engorged females. Females from the second group were not fed on blood, and were anesthetized and weighed in the same manner as the first group was. The amount of blood taken was inferred from the ratio of the weight in the first group to the weight of the second group. The mosquitoes were weighed on an analytical balance (APX-200, Denver instrument^®^, Arvada, CO, USA) according to work done by Dias et al. [7].

### 2.6. Fecundity

This experiment was to determine if there was a significant difference in the number of eggs laid by females fed on different membranes. For *Aedes aegypti*, three days after blood feeding, 30 females per membrane were individually transferred (one female per plate) to 30 Petri dishes (6 cm diameter × 1.5 cm height) adapted for oviposition. The plates were inverted, and the bottom lined internally with filter paper [24]. Three milliliters of rearing water was added to stimulate oviposition. Rearing water was collected from the bowls where larvae were reared, and it contained a low concentration of oxygen, which is a hatching stimulus. Plates were kept in incubators with a constant temperature of 26 °C and relative humidity of 80%. After 24 h, 1 mL of water was added to ensure moisture. After 48 h, the females were removed, and plates containing the filter paper with eggs were left in the incubator for approximately seven days or until the filter paper was completely dry. The number of eggs for each membrane condition was then computed and recorded. *Culex quinquefasciatus* females did not adapt well to individual oviposition. For this reason, three days after the blood meal, three groups of 10 females were transferred to cylindrical cardboard cages measuring 16.5 cm in diameter by 17.5 cm in height, where 50 mL plastic cups containing approximately 45 mL of dechlorinated water were introduced for egg collection. After three days, the total number of eggs from each group of 10 females was computed and recorded. In all conditions, the eggs were counted with the aid of a binocular optical stereomicroscope (M1A, Wild Heerbrugg^®^, Heerbrugg, Switzerland). Fecundity experiments were performed in triplicate, totalizing 90 females per condition per species.

### 2.7. Hatchability

This experiment was to determine the percentage of eggs that hatched according to the type of used membrane. For *Aedes aegypti*, after counting the total number of eggs per female, 10 mL of rearing water was added to each plate to stimulate egg hatching. These plates were maintained in the incubator at a temperature of 26 °C. After 48 h, hatchability was computed per plate, representing the percentage of viable eggs (number of larvae) per female. For *Culex quinquefasciatus*, after the eggs were counted, they were transferred to appropriate bowls for the daily monitoring of the larval hatching rate. The follow-up ended on the first day, when the total number of eggs had hatched, or on the fifth day after the eggs had been counted.

### 2.8. Statistical Analysis

The Kolmogorov–Smirnov test was applied to verify the assumption of normality for the analyzed parameters. After these initial tests, one-way ANOVA and Tukey’s post-test were used to compare the percentage of engorged females, fecundity, hatchability, and weight-increase ratio after the females had been fed through the different types of membranes. Statistical software SPSS 23.0 (IBM SPSS Statistics for Windows, Armonk, NY, USA) was used for the Kolmogorov–Smirnov test, and GraphPad Prism version 6.04 for Windows (GraphPad software, San Diego, CA, USA) was used for one-way ANOVA.

## 3. Results

### 3.1. Artificial Blood Feeding in Aedes aegypti

**Figure 2:**
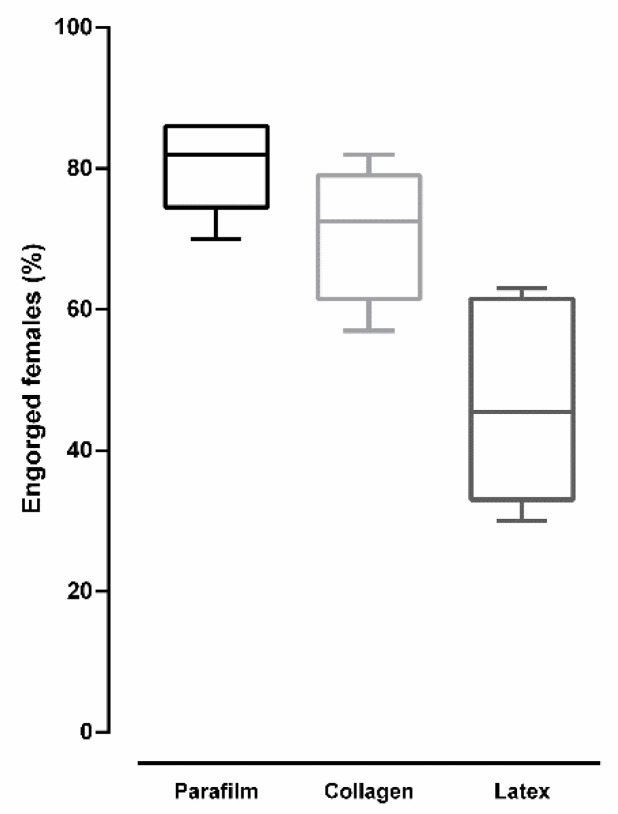
Percentage of engorged *Aedes aegypti* females after artificial blood feeding on different membranes (bars = ±SEM).

Figure 2 shows the percentage of engorged *Aedes aegypti* females after blood feeding on different types of membrane. Overall, there was a significant difference in the percentage of engorged females according to membrane type (ANOVA: *F* (2, 15) = 17.15, *p* = 0.0001). Higher percentages of engorged females were observed when blood-feeding was performed on Parafilm^®^ and collagen. Tukey’s multiple-comparison test revealed no significant difference between Parafilm^®^ and collagen (*p* = 0.28). However, latex was shown to have a significantly smaller percentage of engorged females when compared to those of Parafilm^®^ (*p* < 0.05) and collagen (*p* < 0.05).

**Figure 3:**
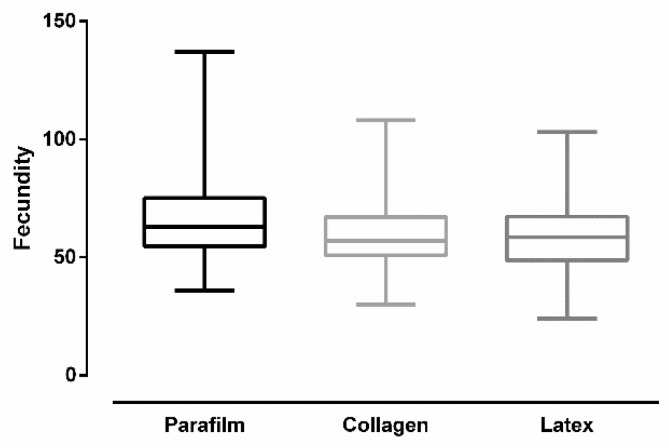
Fecundity of *Aedes aegypti* females after artificial blood feeding on different membranes (bars = ±SEM).

Figure 3 shows the number of eggs per female (fecundity) after *Aedes aegypti* females were fed on different membranes. Overall, there was a significant difference in fecundity according to membrane type (ANOVA: *F* (2, 267) = 7.63, *p* = 0.0006). Females fed on Parafilm^®^ had higher fecundity. Tukey’s multiple-comparison test revealed significant statistical difference between Parafilm^®^ and collagen (*p* < 0.05), and between Parafilm^®^ and latex (*p* < 0.05). No significant difference was observed between collagen and latex (*p* = 0.99).

**Figure 4:**
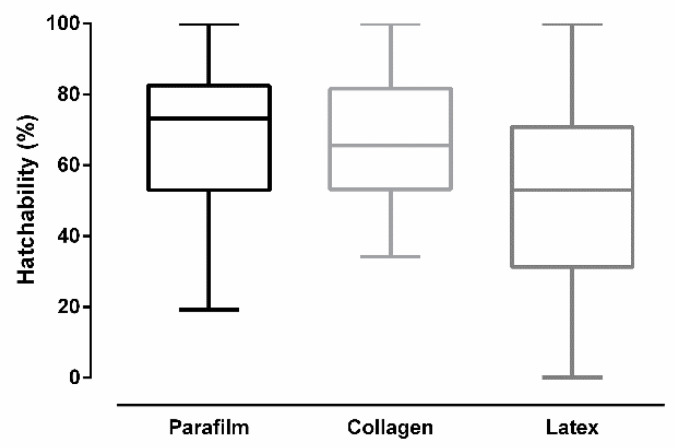
Egg hatchability after *Aedes aegypti* females were artificially blood-fed through different membranes (bars = ±SEM).

Figure 4 shows the percentage of eggs that hatched (hatchability) after *Aedes aegypti* females had been fed on different membranes. Significant differences were found in hatchability according to the used membrane type in artificial feeding (ANOVA: *F* (2, 267) = 17.19, *p* < 0.0001). Tukey’s multiple-comparison test revealed no significant statistical difference between Parafilm^®^ and collagen (*p* = 0.807). The test also revealed that latex was significantly different from Parafilm^®^ and collagen (*p* < 0.0001 for both comparisons).

**Figure 5:**
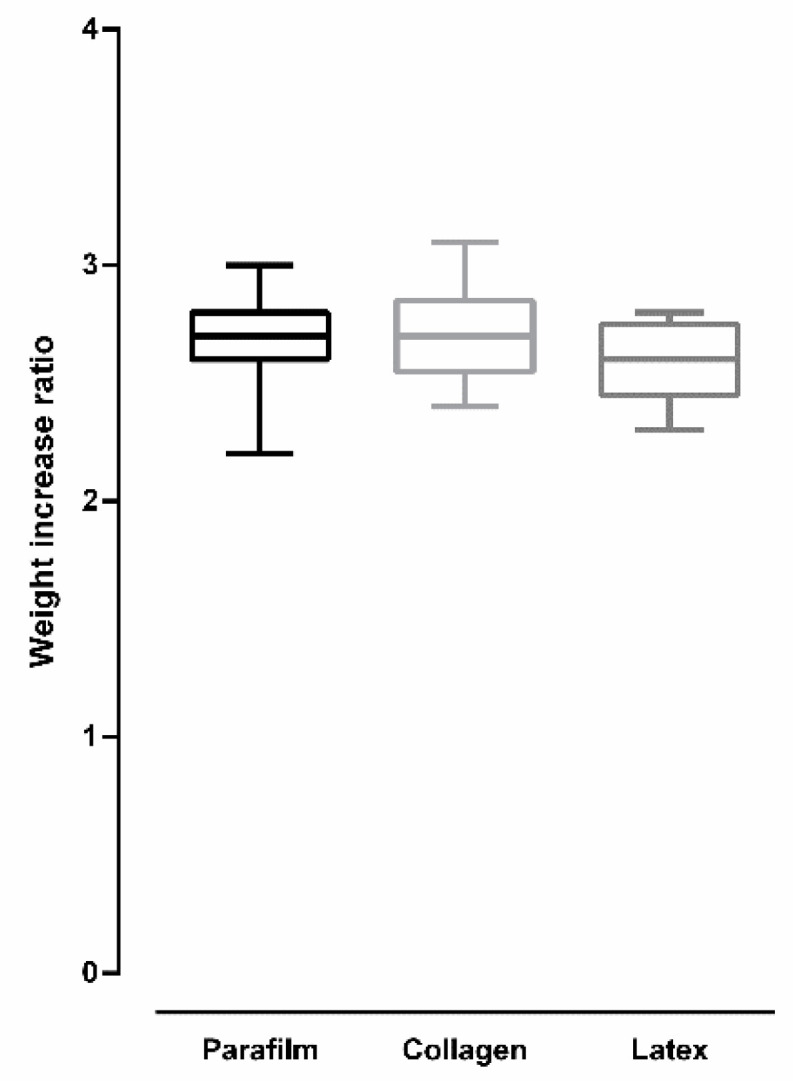
Weight-increase ratio after *Aedes aegypti* females were artificially blood-fed using different membranes. (Bars = ±SEM).

Figure 5 shows the weight-increase ratio after *Aedes aegypti* females were artificially fed on blood using different membranes. For all membranes, weight increase was more than double the original weight of mosquitoes, and no significant differences were observed among the used membranes (ANOVA: *F* (2, 24) = 1.016, *p* = 0.377).

### 3.2. Artificial Blood Feeding in Culex quinquefasciatus

**Figure 6:**
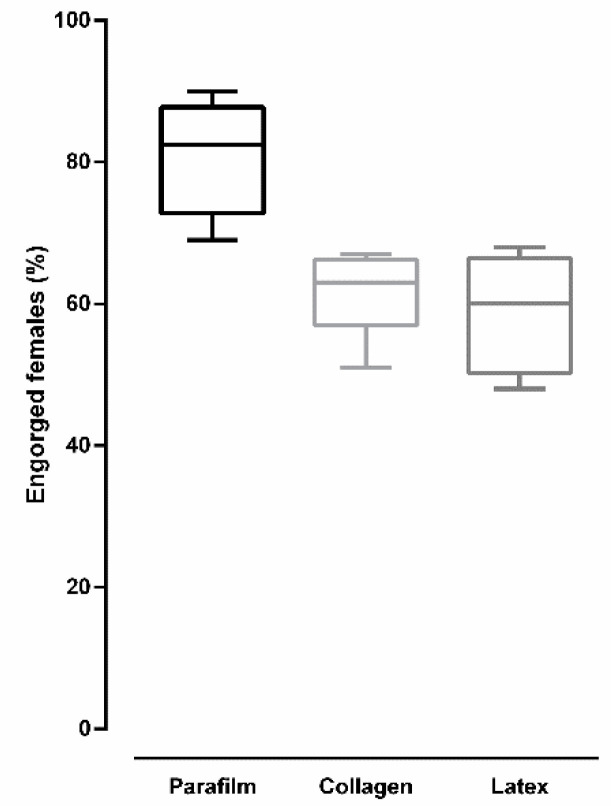
Percentage of engorged *Culex quinquefasciatus* females after artificial blood feeding on different membranes (bars = ±SEM).

Figure 6 shows the percentage of engorged *Culex quinquefasciatus* females after blood feeding on different types of membrane. Overall, there was a significant difference in the percentage of engorged females according to membrane type (ANOVA: *F* (2, 15) = 15.5, *p* = 0.0002). A higher percentage of engorged females was observed when blood-feeding was performed on Parafilm^®^, a value that was significantly different than those values found for collagen and latex (*p* < 0.05 for both comparisons, according to Tukey’s multiple-comparison test). No significant difference was observed between collagen and latex (*p* = 0.812, according to Tukey’s multiple-comparison test).

**Figure 7:**
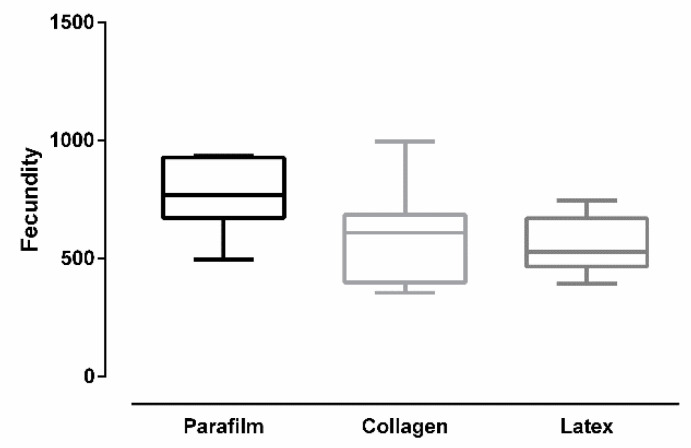
Fecundity of *Culex quinquefasciatus* females after artificial blood feeding on different membranes (bars = ±SEM).

Figure 7 shows the number of eggs per female (fecundity) after *Culex quinquefasciatus* females were artificially blood-fed through different membranes. Overall, there was a significant difference in fecundity according to membrane type (ANOVA: *F* (2, 24) = 4.96, *p* = 0.0158). Females fed on Parafilm^®^ had higher fecundity. Tukey’s multiple-comparison test revealed a significant statistical difference between Parafilm^®^ and latex (*p* < 0.05). The test revealed no significant difference between Parafilm^®^ and collagen (*p* = 0.0648), and between collagen and latex (*p* = 0.8178).

**Figure 8:**
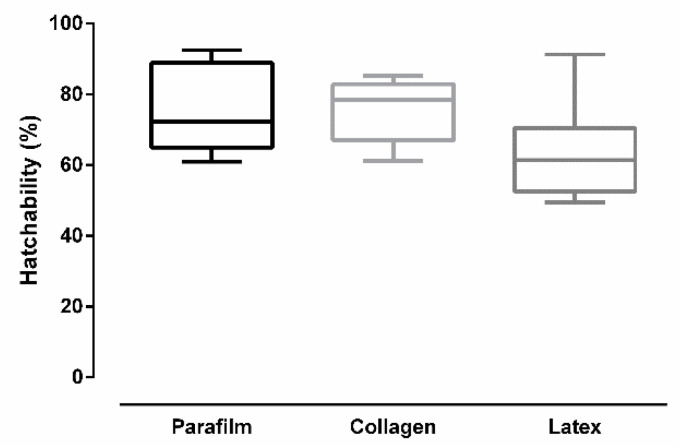
Egg hatchability after *Culex quinquefasciatus* females were artificially blood-fed through different membranes (bars = ±SEM).

Figure 8 shows the percentage of eggs that hatched (hatchability) after *Culex quinquefasciatus* females had been fed on different membranes. Significant differences were found in hatchability according to the used membrane type in artificial feeding (ANOVA: *F* (2, 24) = 3.932, *p* = 0.0333). Tukey’s multiple-comparison test revealed no significant statistical difference between Parafilm^®^ and collagen (*p* = 0.9633), and between collagen and latex (*p* = 0.077). The test also revealed that latex was significantly different than Parafilm^®^ (*p* = 0.0449).

**Figure 9:**
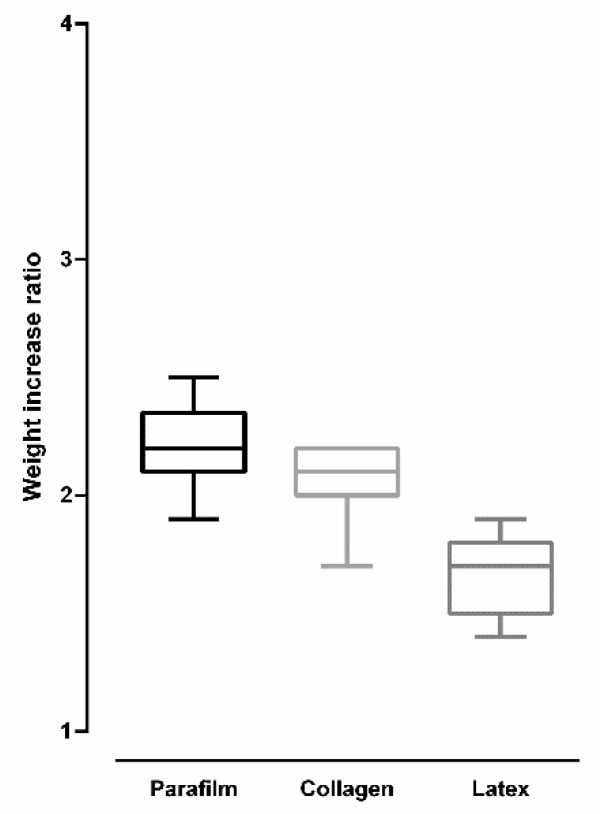
Weight-increase ratio after *Culex quinquefasciatus* females were artificially blood-fed using different membranes (bars = ±SEM).

Figure 9 shows the weight-increase ratio after *Culex quinquefasciatus* females were artificially fed on blood using different membranes. Overall, there was a significant difference in weight-increase ratio according to the used membrane type (ANOVA: *F* (2, 24) = 22.99, *p* < 0.0001). Females fed on Parafilm^®^ and collagen showed an increase that was more than double the original weight before feeding. Tukey’s multiple-comparison test revealed no significant statistical difference between Parafilm^®^ and collagen (*p* = 0.154). Females fed on latex showed a weight increase that was less than double the original weight before feeding. Tukey’s multiple-comparison test revealed a significant statistical difference between Parafilm^®^ and latex, and between collagen and latex (*p* < 0.05 for both comparisons).

Tables containing raw data are available in Supplementary Materials (Tables S1–S4).

## 4. Discussion

Many studies have been conducted using membranes for the artificial blood feeding of insects. Rutledge et al. [18] described and tested a membrane feeder designed specifically for experimental mosquito infection. In this system, hematophagous insects could be fed through membranes.

The system consisted of conical segments made of jacketed glass, connected by an external circuit filled with water heated at 38 ± 1 °C, which circulated through the glass. Membranes containing blood were placed at the glass base. Investigated parameters included type of diet, percentage of feeding, phage stimulants, volatile substances associated with blood, species characteristics, egg production, viability, membrane types, and attraction. The authors concluded that natural membranes, mainly chick skin, had a better utility than that of artificial membranes. Several other authors used the apparatus developed by Rutledge et al. [18,25,26,27,28]. Later, Novak et al. [25] tested the efficiency of rat skin, quail skin, sheep intestine, and latex condoms in artificial mosquito feeding. The authors used defibrinated rabbit blood and the same system as that used by Rutledge et al. [18]. Pothikasikor et al. [26] compared direct *Aedes aegypti* feeding (in human patients) and artificial feeding using blood from patients infected with *Wuchereria bancrofti*. The authors used membranes from chicken, mouse, and swine, and the system described by Rutledge et al. [18]. They concluded that, among the membranes, chicken skin was the best for mosquito feeding. Lyski et al. [27] performed artificial feeding in *Aedes aegypti* and *Aedes albopictus* using defibrinated bovine blood. The authors compared sheep-skin membranes with sausage-casing membranes made from bovine collagen using the same feeding system as that used by previous authors. They noticed that both species preferred the bovine membranes. Like Rutledge et al. [18], the authors found that animal-derived membranes are more efficient in the artificial feeding of mosquitoes. Normal feeding occurs when their diet is covered with a suitable membrane that the insect can pierce deploying its mouthparts, as it would when feeding on a normal host [29].

Nasirian and Ladonni [28] fed *Anopheles stephensi* with whole human blood for three consecutive generations using Parafilm^®^ as a membrane. The highest percentage of fed females was 90.9%, with an average of 75.6%. In another study, Dias et al. [7] fed three species of Culicidae with citrated rabbit blood using a Parafilm^®^ membrane. The percentages of fed females for *Aedes aegypti*, *Culex quinquefasciatus*, and *Anopheles aquasalis* were 98%, 48% and 96%, respectively. In the present work, after using a Parafilm^®^ membrane for artificial feeding, averages of 80.3% of engorged females for *Aedes aegypti* and 80.8% of engorged females for *Culex quinquefasciatus* were obtained. This shows that, despite the preference of mosquitoes for animal membranes, when Parafilm^®^ is well-prepared, it becomes very efficient in artificial feeding. However, we observed that the wider that Parafilm^®^ is stretched, the greater the chance of rupture. This is not desirable since such an incident, in addition to compromising the running experiment, messes up the bench, cages, and utensils. It also compromises the operational routine of the laboratory, especially when the goal is the maintenance of colonies on a large scale.

Females of *Culex quinquefasciatus* are considered to be more reluctant to feed on membranes than females of *Aedes aegypti* are [7,25,30]. Our results showed that this reluctance depends on the type of membrane used, as Parafilm^®^ showed a high percentage of fed females, a value that was significantly higher than values found for collagen and latex.

For *Aedes aegypti*, fecundity was significantly lower when artificial feeding had been performed using collagen and latex. The reason for this deficit is not clear. Latex was shown to be less effective in artificial feeding [21,25] for mosquitoes but not triatomines [31]. It would be interesting to investigate if some chemical component present in the latex and collagen membranes influenced the number of the eggs.

For both species, there was no significant difference in hatchability when artificial feeding was performed with Parafilm^®^ and collagen. The females of both species fed on latex membranes showed a significant decrease in hatchability. If some chemical component present in latex affects fecundity, it may also affect hatchability.

Weight-increase ratio was the only parameter that had not been affected by the type of membrane used to feed *Aedes aegypti* females. Although the latex membrane negatively affected the other parameters evaluated in this species, it was not able to influence female behavior for the amount of ingested blood. Weight-increase ratio was significantly lower when *Culex quinquefasciatus* females had been fed on latex when compared with those fed with Parafilm^®^ and collagen. This result indicates that, besides negatively affecting the other parameters evaluated in this species, the latex membrane could also negatively modulate the females’ feeding behavior.

The artificial-feeding methodology used in this work is already standardized and widely used in colonies of several hematophagous insects kept in the laboratory. The choice of a suitable membrane is of great importance for the success of artificial blood-feeding in mosquitoes. Among the membranes evaluated in the present work, collagen was almost as effective as the Parafilm^®^ membrane, especially for *Aedes aegypti*, with the advantages of being more resistant and easier to handle. Another great advantage of collagen in relation to Parafilm^®^ is the possibility of reuse, since the required temperature for artificial feeding (38 °C) weakens Parafilm^®^ and could lead to its rupture, which was not observed for collagen membrane. Hence, the collagen membrane allows for the feeding of cages in sequence without the need for interruption for their replacement, optimizing the feeding process and, consequently, the laboratory operating routine. Although collagen shows some advantages over Parafilm^®^, one might still choose the latter for artificial blood feeding, as it showed a better performance considering the analyzed feeding and reproduction parameters. Collagen and Parafilm^®^, associated with more modern methods, can produce even better results in relation to success in feeding, fecundity, and egg-hatching rate for large-scale mosquito production, as it is of great importance to avoid the use of animals for this purpose.

## 5. Conclusions

The assessment of Parafilm^®^, collagen, and latex for artificial blood feeding revealed that latex should be avoided, as it compromises some parameters related to the feeding and reproduction of two mosquito species. Collagen is a good alternative to Parafilm^®^, as it performed almost as efficiently as Parafilm^®^ did, with the advantages of being more resistant and easier to handle. However, Parafilm^®^ could be the first choice, as it did not show poorer performance when compared with the two other evaluated membranes in any aspect.

## Supplementary Materials

The following are available online at https://www.mdpi.com/2075-4450/12/1/15/s1, Table S1: comparison of fecundity and percentage of hatchability for *Aedes aegypti* lab strain fed through Parafilm^®^, collagen membrane, and latex gloves; Table S2: comparison of fecundity and percentage of hatchability for *Culex quinquefasciatus* lab strain fed through Parafilm^®^, collagen membrane, and latex gloves; Table S3: number of engorged females and weight for *Aedes aegypti* lab strain fed through Parafilm^®^, collagen membrane, and latex gloves; Table S4: number of engorged females and weight for *Culex quinquefasciatus* lab strain fed through Parafilm^®^, collagen membrane, and latex gloves.
