# Supplementary material for: Assessment of Synthetic Membranes for Artificial Blood Feeding of Culicidae
Source: Insects. 2020 Dec 29;12(1):15. doi: 10.3390/insects12010015 (PMC7824735; doi:10.3390/insects12010015)
Supplement: Supplementary file 1 [file insects-12-00015-s001.pdf]

Supplementary Table S1: Comparison of fecundity and percentage of hatchability for *Ae. aegypti* lab strain fed through Parafilm®, collagen membrane, and latex gloves.

	Aedes aegypti fecundity and hatchability									
		Parafilm®			Collagen			Latex		
	Mosquito	Eggs	Hatchability		Eggs	Hatchability		Eggs	Hatchability	
			Number	%		Number	%		Number	%
Experiment 1	01	74	43	58,1	48	27	56,3	80	38	47,5
	02	84	35	41,7	71	27	38,0	71	52	73,2
	03	61	38	62,3	43	23	53,5	70	45	64,3
	04	65	32	49,2	85	50	58,8	68	22	32,4
	05	68	56	82,4	44	26	59,1	64	2	3,1
	06	51	25	49,0	58	31	53,4	50	50	100,0
	07	68	24	35,3	60	37	61,7	63	30	47,6
	08	61	45	73,8	64	52	81,3	49	14	28,6
	09	69	46	66,7	53	34	64,2	82	25	30,5
	10	58	16	27,6	59	36	61,0	92	20	21,7
	11	75	20	26,7	52	28	53,8	61	9	14,8
	12	91	39	42,9	46	36	78,3	40	25	62,5
	13	56	52	92,9	38	20	52,6	85	22	25,9
	14	71	35	49,3	70	43	61,4	53	28	52,8
	15	69	48	69,6	63	38	60,3	26	25	96,2
	16	62	57	91,9	67	29	43,3	75	11	14,7
	17	73	14	19,2	52	27	51,9	58	21	36,2
	18	97	62	63,9	61	24	39,3	89	30	33,7
	19	82	41	50,0	58	23	39,7	58	41	70,7
	20	109	54	49,5	57	34	59,6	59	22	37,3
	21	56	44	78,6	59	39	66,1	51	18	35,3
	22	104	45	43,3	44	23	52,3	60	4	6,7
	23	58	30	51,7	41	23	56,1	60	14	23,3
	24	69	36	52,2	51	37	72,5	79	41	51,9
	25	81	38	46,9	49	31	63,3	62	33	53,2
	26	36	18	50,0	42	37	88,1	42	23	54,8
	27	45	24	53,3	54	23	42,6	70	7	10,0
	28	36	36	100,0	38	13	34,2	48	42	87,5
	29	52	27	51,9	30	16	53,3	83	28	33,7
	30	51	23	45,1	43	28	65,1	30	20	66,7
Experiment 2	01	72	33	45,8	67	25	37,3	65	41	63,1
	02	46	37	80,4	57	28	49,1	55	20	36,4
	03	92	52	56,5	54	25	46,3	42	25	59,5
	04	59	55	93,2	49	26	53,1	52	35	67,3
	05	75	65	86,7	73	61	83,6	24	2	8,3
	06	55	25	45,5	44	31	70,5	32	2	6,3
	07	90	85	94,4	62	52	83,9	68	30	44,1

	08	84	67	79,8	59	51	86,4	53	27	50,9
	09	69	58	84,1	69	62	89,9	62	26	41,9
	10	60	46	76,7	55	22	40,0	51	35	68,6
	11	60	48	80,0	53	42	79,2	67	15	22,4
	12	45	45	100,0	90	67	74,4	47	3	6,4
	13	50	28	56,0	45	23	51,1	47	6	12,8
	14	62	48	77,4	56	25	44,6	52	20	38,5
	15	79	61	77,2	59	40	67,8	75	34	45,3
	16	54	32	59,3	63	45	71,4	57	35	61,4
	17	64	56	87,5	62	23	37,1	44	24	54,5
	18	62	35	56,5	57	35	61,4	72	37	51,4
	19	53	32	60,4	68	56	82,4	40	30	75,0
	20	61	43	70,5	54	43	79,6	38	12	31,6
	21	45	31	68,9	51	21	41,2	37	1	2,7
	22	60	58	96,7	43	20	46,5	53	28	52,8
	23	51	50	98,0	40	23	57,5	56	32	57,1
	24	48	30	62,5	61	26	42,6	64	5	7,8
	25	60	54	90,0	70	49	70,0	64	56	87,5
	26	100	83	83,0	68	53	77,9	42	30	71,4
	27	67	50	74,6	61	54	88,5	52	2	3,8
	28	82	73	89,0	47	38	80,9	65	25	38,5
	29	70	56	80,0	43	16	37,2	50	8	16,0
	30	51	39	76,5	52	29	55,8	32	0	0,0
Experiment 3	01	90	64	71,1	54	50	92,6	76	50	65,8
	02	75	43	57,3	91	68	74,7	65	29	44,6
	03	73	22	30,1	54	51	94,4	50	31	62,0
	04	66	49	74,2	61	61	100,0	40	22	55,0
	05	51	47	92,2	57	56	98,2	55	45	81,8
	06	64	48	75,0	51	44	86,3	57	27	47,4
	07	55	21	38,2	87	76	87,4	63	58	92,1
	08	55	53	96,4	71	64	90,1	51	28	54,9
	09	137	91	66,4	57	57	100,0	36	5	13,9
	10	71	58	81,7	58	42	72,4	63	40	63,5
	11	107	79	73,8	78	65	83,3	65	57	87,7
	12	47	39	83,0	90	77	85,6	67	36	53,7
	13	48	28	58,3	108	86	79,6	35	4	11,4
	14	51	35	68,6	74	66	89,2	66	57	86,4
	15	61	45	73,8	56	41	73,2	62	30	48,4
	16	54	46	85,2	73	58	79,5	60	60	100,0
	17	63	53	84,1	49	37	75,5	37	37	100,0
	18	109	97	89,0	56	50	89,3	80	80	100,0
	19	58	47	81,0	60	53	88,3	66	49	74,2
	20	86	63	73,3	71	43	60,6	93	83	89,2
	21	83	61	73,5	56	37	66,1	48	32	66,7
	22	67	49	73,1	79	70	88,6	68	65	95,6

	23	74	48	64,9	49	35	71,4	45	26	57,8
	24	58	42	72,4	50	32	64,0	57	39	68,4
	25	68	56	82,4	74	46	62,2	61	56	91,8
	26	63	59	93,7	90	48	53,3	90	72	80,0
	27	43	40	93,0	52	37	71,2	82	64	78,0
	28	46	36	78,3	59	48	81,4	103	79	76,7
	29	76	59	77,6	53	28	52,8	52	50	96,2
	30	61	38	62,3	57	47	82,5	55	35	63,6

Supplementary Table S2: Comparison of fecundity and percentage of hatchability for *Cx. quinquefasciatus* lab strain fed through Parafilm®, collagen membrane, and latex gloves.

	Culex quinquefasciatus fecundity and hatchability									
	Parafilm®				Collagen			Latex		
	Group (10 mosquitoes)	Eggs	Hatchability Number	Hatchability %	Eggs	Hatchability Number	Hatchability %	Eggs	Hatchability Number	Hatchability %
Experiment 1	01	925	564	61,0	597	365	61,1	746	518	69,4
	02	935	601	64,3	691	589	85,2	677	484	71,5
	03	932	612	65,7	996	656	65,9	665	366	55,0
Experiment 2	01	817	586	71,7	419	350	83,5	479	294	61,4
	02	742	662	89,2	355	242	68,2	535	265	49,5
	03	498	360	72,3	381	287	75,3	393	359	91,3
Experiment 3	01	603	535	88,7	682	553	81,1	477	271	56,8
	02	768	679	88,4	610	502	82,3	528	336	63,6
	03	749	693	92,5	635	498	78,4	456	229	50,2

Supplementary Table S3: Number of engorged females and weight for *Ae. aegypti* lab strain fed through, Parafilm®, collagen membrane, and latex gloves.

Number of engorged *Aedes aegypti* females and weight of groups of 10 females after artificial blood feeding through membranes

	Number of engorged females						Weight (g)								
	Experiment 1		Experiment 2		Experiment 3										
Membrane	Cage 1	Cage 2	Cage 1	Cage 2	Cage 1	Cage 2	Experiment 1			Experiment 2			Experiment 3		
Not fed	86	86	76	70	79	85	0,0110	0,0128	0,0121	0,0129	0,0137	0,0140	0,0121	0,0120	0,0126
Parafilm®	75	70	63	57	78	82	0,0335	0,0285	0,0312	0,0338	0,0377	0,0397	0,0343	0,0324	0,0322
Collagen	63	61	34	30	49	42	0,0338	0,0376	0,0332	0,0357	0,0374	0,0336	0,0340	0,0315	0,0320
Latex	86	86	76	70	79	85	0,0308	0,0316	0,0332	0,0360	0,0358	0,0328	0,0310	0,0308	0,0298

Supplementary Table S4: Number of engorged females and weight for *Cx. quinquefasciatus* lab strain fed through Parafilm®, collagen membrane, and latex gloves.

Number of engorged *Culex quinquefasciatus* females and weight of groups of 10 females after artificial blood feeding through membranes															
	Number of engorged females						Weight (g)								
	Experiment 1		Experiment 2		Experiment 3										
Membrane	Cage 1	Cage 2	Cage 1	Cage 2	Cage 1	Cage 2	Experiment 1			Experiment 2			Experiment 3		
Not fed	90	83	87	69	82	74	0,0190	0,0195	0,0186	0,0228	0,0208	0,0206	0,0222	0,0231	0,0196
Parafilm®	62	51	67	66	64	59	0,0457	0,0484	0,0429	0,0506	0,0450	0,0455	0,0449	0,0436	0,0431
Collagen	66	64	68	51	56	48	0,0404	0,0398	0,0408	0,0459	0,0439	0,0455	0,0438	0,0396	0,0434
Latex	90	83	87	69	82	74	0,0354	0,0296	0,0334	0,0398	0,0370	0,0350	0,0343	0,0327	0,0346
